# Luteolin exerts anti‐tumour immunity in hepatocellular carcinoma by accelerating CD8 ^+^ T lymphocyte infiltration

**DOI:** 10.1111/jcmm.18535

**Published:** 2024-09-12

**Authors:** Shijiao Cai, Yidan Gou, Yanyan Chen, Xiaoran Hou, Jing Zhang, Chongwen Bi, Peng Gu, Miao Yang, Hanxu Zhang, Weilong Zhong, Hengjie Yuan

**Affiliations:** ^1^ Department of Pharmacy Tianjin Medical University General Hospital Tianjin China; ^2^ Tianjin Key Laboratory of Digestive Diseases, Department of Gastroenterology and Hepatology Tianjin Institute of Digestive Diseases, Tianjin Medical University General Hospital Tianjin China

**Keywords:** CD8^+^ T lymphocyte infiltration, hepatocellular carcinoma, high‐throughput sequencing, luteolin, PD‐1 inhibitor

## Abstract

Luteolin, a commonly used traditional Chinese medicine, has been utilized for several decades in the treatment of hepatocellular carcinoma (HCC). Previous research has demonstrated its anti‐tumour efficacy, but its underlying mechanism remains unclear. This study aimed to assess the therapeutic effects of luteolin in H22 tumour‐bearing mice. luteolin effectively inhibited the growth of solid tumours in a well‐established mouse model of HCC. High‐throughput sequencing revealed that luteolin treatment could enhance T‐cell activation, cell chemotaxis and cytokine production. In addition, luteolin helped sustain a high ratio of CD8^+^ T lymphocytes in the spleen, peripheral blood and tumour tissues. The effects of luteolin on the phenotypic and functional changes in tumour‐infiltrating CD8^+^ T lymphocytes were also investigated. Luteolin restored the cytotoxicity of tumour‐infiltrating CD8^+^ T lymphocytes in H22 tumour‐bearing mice. The CD8^+^ T lymphocytes exhibited intensified phenotype activation and increased production of granzyme B, IFN‐γ and TNF‐α in serum. The combined administration of luteolin and the PD‐1 inhibitor enhanced the anti‐tumour effects in H22 tumour‐bearing mice. Luteolin could exert an anti‐tumour immune response by inducing CD8^+^ T lymphocyte infiltration and enhance the anti‐tumour effects of the PD‐1 inhibitor on H22 tumour‐bearing mice.

## 1 INTRODUCTION

Hepatocellular carcinoma (HCC) is a prevalent liver malignant tumour and ranks sixth in terms of global incidence and second in terms of global mortality.^1^ Our evolving understanding of tumorigenesis reveals that HCC is predominantly induced by a series of chronic liver diseases, including liver cirrhosis, hepatitis B virus/hepatitis C virus infection and fatty liver disease.^2^ The tumour microenvironment (TME) contains abundant immune, stromal and tumour cells, and it plays a key role in tumour heterogeneity and malignant progression.^3^ The complexity of tumours and the immune evasion mechanisms within TME are among the primary reasons for the development of resistance to immunotherapy. Other factors contributing to this resistance include the lack of effective neoantigens in tumours, impaired tumour antigen presentation, inadequate infiltration of immune cells into the tumour, compromised interferon‐gamma (IFN‐γ) signalling, metabolic and inflammatory mediators, immunosuppressive cells, altered immune checkpoints, severe T‐cell exhaustion and epigenetic alterations in T cells.^4^

Tumour‐infiltrating lymphocytes (TILs), represented by T, B and NK cells, are major type of infiltrating immune cells. T lymphocytes can be classified into the following subtypes in accordance with their cell surface markers: CD4^+^ T helper lymphocytes, CD45RO^+^ memory T cells, CD8^+^ cytotoxic T lymphocytes (CTLs) and FOXP3^+^ regulatory cells. Elevated levels of CD8^+^ T lymphocytes in the tumour stroma and nest have been associated with improved overall survival of patients with HCC.^5^, ^6^ The immune microenvironment that sustains tumour survival consists of various infiltrating immune cells and cytokines. Among them, CD8^+^ T lymphocytes are the effector cells in the tumour immune response and the core force in eliminating tumours. Under normal circumstances, CD8^+^ T lymphocytes can directly destroy cancer cells by releasing cytotoxic granules containing perforin and granzyme B.^7^ Alternatively, they can indirectly induce cancer cell death by releasing substances such as IFN‐γ^8^ and tumour necrosis factor‐alpha (TNF‐α).^9^ These cells either engage in direct contact or secrete factors that interact with other cytokines and chemokines to regulate and maintain tumour growth through autocrine and paracrine mechanisms.

The potential anti‐tumour effects of flavonoids on various tumours have gained attention.^10^, ^11^ Increasing evidence has highlighted luteolin as an anti‐tumour agent against various types of tumours, such as lung,^12^ breast,^13^ glioblastoma,^14^ prostate,^15^ and colon^16^ cancers. Most flavonoids produce outstanding therapeutic outcomes when they are combined with anti‐PD‐1/PD‐L1 antibodies. Apigenin, a bioavailable flavonoid found in celery, has been reported to inhibit the high‐level expression of PD‐L1 induced by IFN‐γ by restricting the phosphorylation of STAT1 in breast cancer and melanoma cells.^17^ Puerarin, a flavonoid extracted from kudzu root, can sensitize anti‐PD‐L1 antibody therapy by regulating the reactive oxygen species (ROS) level to decrease the number of cancer‐associated fibroblasts.^18^

Luteolin (3,4,5,7‐tetrahydroxy flavone) is a natural flavonoid that is extensively present in many plant species. It is particularly abundant in fruits and vegetables, such as celery, chrysanthemum flowers, sweet bell peppers, carrots, onion leaves, broccoli and parsley.^19^ Luteolin hampers the progression of carcinogenesis through multiple mechanisms. Luteolin exerts an anti‐tumour effect by activating the PI3K‐Akt signalling pathway in antigen‐presenting cells (APCs), leading to the induction of APC activation, enhancement of CTL responses, suppression of T‐cell exhaustion and improvement of the immune microenvironment in melanoma.^20^ Cao et al. reported that luteolin treatment induces a substantial G0−/G1‐phase arrest and decreases the expression of the anti‐apoptotic protein BCL‐2, which controls the intrinsic mitochondrial pathway and is connected with mitochondrial‐mediated signals through a reduction in the mitochondrial membrane potential and the release of pro‐apoptotic proteins. Luteolin‐activated caspase‐8, which controls the extrinsic pathway, in turn trigger caspase‐3 in human HepG2 cells.^21^ Luteolin inhibits VEGF‐/bFGF‐induced angiogenesis in vitro by inhibiting matrix‐degrading proteases.^22^ It causes cell death induction and tumour reduction in HepG2 tumour‐bearing mice. It also suppresses the NF‐κB DNA‐binding activity and facilitates the release of ROS.^23^

Increasing attention has been paid to the use of luteolin to treat cancers because of its potential to improve antitumor immunity.^24^ However, the anti‐tumorigenesis of luteolin combined with the PD‐1 inhibitor in HCC remains unclear. Hence, we aimed to investigate the therapeutic outcomes of luteolin combined with anti‐PD‐1 antibody in treating HCC.

## 2 MATERIALS AND METHODS

### 2.1 Experimental animals and cells

Mouse HCC cell line H22 was purchased from KeyGen Biotech (Nanjing, China) and cultured using RPMI‐1640 containing 10% fetal bovine serum and 1% penicillin–streptomycin at 5% CO_2_ and 37°C. Female BALB/c mice (aged 6–8 weeks; Beijing Sbeifu Biotechnology, Beijing, China) were used as experimental animals and housed in a specific pathogen‐free animal facility under controlled environmental conditions with a 12 h light–dark cycle.

### 2.2 Establishment of subcutaneous H22 tumour‐bearing mice and in vivo experiment

The H22 cells were subcutaneously injected into the BALB/c mice (2 × 10^6^ cells in 100 μL PBS), which were randomly divided into four groups (*n* = 4) as follows: control (0.5% carboxymethyl cellulose sodium [CMC‐Na]), 50 mg/kg luteolin (Meilunbio), 100 mg/kg luteolin and 200 mg/kg luteolin. All treatments were administered strictly once daily via gavage for 21 days in a volume of 0.2 mL. Tumour volume and body weight were measured every 4 days. Tumour volume was further evaluated using the formula *V* = length × width^2^/2. All animal experiments were performed with the approved protocols of the Experimental Animal Welfare Ethics Committee of Tianjin Medical University General Hospital.

### 2.3 High‐throughput sequencing

Tumour tissues were collected from the control and 200 mg/kg luteolin groups. Total RNA was extracted using the Trizol reagent (Thermo Fisher Scientific). Its concentration was measured with a Qubit fluorometer (Thermo Fisher Scientific) and its quality was checked by a 2100 Bioanalyzer (Agilent Technologies, Santa Clara, *CA*). An RNA library was prepared using the TruSeq RNA sample preparation kit V2 (Vazyme Biotech) with 1 μg of total RNA as the input following the manufacturer's instructions. The library was quantified with the Kapa Library quantification kit (Kapa Biosystems, Wilmington, MA) using an ABI 2720 thermal cycler (Thermo Fisher Scientific). The final library was subjected to high‐throughput sequencing on the Illumina NovaSeq 6000 platform using a 2 × 150 bp paired‐end sequencing mode. The sequencing data were deposited in the Gene Expression Omnibus and the accession number is GSE243341 (https://www.ncbi.nlm.nih.gov/geo/query/acc.cgi?acc=GSE243341).

### 2.4 Bioinformatics analysis

The differential expression between the control and luteolin groups was analysed using DESeq2‐v1.10.1. Genes with a *p* value <0.05 and fold change >2 were identified as up‐regulated, and those with a *p* value <0.05 and fold change <−2 were identified as down‐regulated. Volcano map and hierarchical clustering were generated with the R package pheatmap and R package volcano, respectively. The functions of up‐regulated and down‐regulated genes were implemented using online tools (http://cloud.geneskybiotech.com/#/tools/all/go_kegg_enrichment).

### 2.5 Real‐time quantitative reverse transcription–polymerase chain reaction

Real‐time quantitative reverse transcription–polymerase chain reaction (qRT‐PCR) was used to detect the transcription level of CD8^+^ T‐cell infiltration (Cd3e, Cd8a, Ccl5 and Ccl21), CD8^+^ T‐cell activation (Gzmb, Ifng and Tnf) and apoptosis (Casp3, Casp8, Casp9, Bax and Bcl2) mRNA in tumour tissues. Total RNA was extracted from cells and tissues by using TRIzol™ (Invitrogen, Carlsbad, CA, USA) and gene expression was analysed using CFX384 (Bio‐Rad, Hercules, CA, USA). The transcriptional levels were normalized by β‐actin. The primer sequences are shown in Table S1.

### 2.6 Isolation of mononuclear cells from the spleen, peripheral blood and tumour tissues

Suitably sized spleens were delicately ground by making them pass through a sterile 70‐gauge steel mesh. The cells were subsequently gathered via centrifugation at 2000 rpm for 10 min. The lymphocytes were prepared using red blood cell lysis buffer (Solarbio, Beijing), followed by washing with PBS to obtain a suspension of lymphocytes. A peripheral blood sample was obtained from the retrobulbar venous plexus, palced into an anticoagulant tube and utilized to isolate peripheral T cells by using a mouse peripheral blood lymphocyte separation kit (TBD Company, Tianjin, China) following the manufacturer's instructions. For the analysis of tumour‐infiltrating T lymphocytes, the tumours were fragmented into small pieces and digested using 25 μg/mL IV collagen and 150 U/mL DNase I. The cell suspension gently passed through a 70‐gauge steel mesh and the TILs were isolated through gradient centrifugation in PBS by using a 30%–70% Percoll (Solarbio, Beijing) solution. The gradient was then subjected to centrifugation at 1000 rpm for 10 min. The interphase containing the TILs was collected and washed two times with PBS.

### 2.7 Flow cytometry

CD3e, an element of the T‐cell receptor complex, is located on the surface of mature T cell. CD8α, a surface protein, is found on a subset of T cells referred to as cytotoxic T cells (also known as CD8^+^ T cells). In this study, CD8^+^ T lymphocytes were transferred into FACS tubes and stained with the following fluorescence‐conjugated monoclonal antibodies: anti‐mouse fluorescein isothiocyanate‐conjugated CD3e, anti‐mouse allophycocyanin‐conjugated CD8α and corresponding isotype controls. Data were acquired with an LSRFortessa™ cell analyser (Cat No. 647780P3, BD) and analysed with FlowJo software (version 10.8).

### 2.8 Haematoxylin and eosin staining

The mouse tumour tissues were sliced into 1.0 cm × 1.0 cm × 0.3 cm dimensions, fixed in 4% paraformaldehyde for 24 h, dehydrated, embedded in paraffin and cut into 4 μm thick sections. After dewaxing and rehydration, the sections were stained with haematoxylin for 5 min, counterstained with eosin for 5 min and observed under a light microscope. The nuclei appeared purplish blue and the cytoplasm was pink. Finally, the haematoxylin‐and‐eosin‐stained sections were scanned and examined using Pannoramic MIDI.

### 2.9 Immunohistochemistry

The tumours were embedded in paraffin and cut into serial transverse sections (5 μm). The slides were deparaffinized and dehydrated before immunohistochemistry (IHC) was performed. After blocking with 10% normal goat serum for 20 min, the sections were incubated with primary antibodies, including anti‐CD8α (1:200; CST) and anti‐cleaved caspase‐3 (1:200; CST) at 4°C overnight. Then, the sections were incubated with horseradish peroxidase‐conjugated secondary antibodies for 1 h at room temperature. Photographs were scanned and examined using Pannoramic MIDI. The expression levels of CD8α and cleaved caspase‐3 were analysed by two independent investigators.

### 2.10 ELISA

The blood sample of the mice collected from the retrobulbar venous plexus was placed in a red blood collection vessel and put at room temperature for 1 h. Serum was obtained after centrifugation at 3000 rpm for 10 min. The levels of granzyme B, IFN‐γ and TNF‐α were quantified by ELISA (Gelatins, Shanghai, China) in accordance with the manufacturer's instructions.

### 2.11 Effect of luteolin combined with the PD‐1 inhibitor in vivo

For the combination experiment, H22 cells were subcutaneously injected into BALB/c mice (2 × 10^6^ cells in 100 μL PBS). When the tumour volume reached ∼100 mm^3^, the mice were randomly distributed into the following groups (*n* = 4): control, luteolin, PD‐1 inhibitor and a combination of luteolin and PD‐1 inhibitor. The control group was treated by gavage with the vehicle alone (0.5% CMC‐Na) for 17 days. The luteolin group was treated by gavage with luteolin (100 mg/kg daily) for 17 days. The PD‐1 inhibitor group was intraperitoneally given PD‐1 inhibitor (clone 29F.1A12™, Bio X Cell) on days 7, 11, 15, 19 and 23 (100 μg/injection). The combination of luteolin and PD‐1 inhibitor group was treated by gavage with luteolin (100 mg/kg daily) and intraperitoneally given the PD‐1 inhibitor on days 7, 11, 15, 19 and 23 (100 μg/injection). Tumour volume and body weight were measured every 4 days. Tumour volume was further evaluated using the formula *V* = length × width^2^/2.

### 2.12 Statistical analysis

Data were presented as mean ± SD and visualized using GraphPad software (version 10.0.2, Inc., La Jolla, CA, USA). Student's *t* test was performed to evaluate the statistical significance between two independent groups. One‐way ANOVA was utilized to compare multiple groups of data. Correlation analysis was performed using the Spearman algorithm of cor. test function in R software. *p* < 0.05 indicated statistical significance.

## 3 RESULTS

### 3.1 Luteolin exerts a dose‐dependent anti‐tumour effect

**FIGURE 1:**
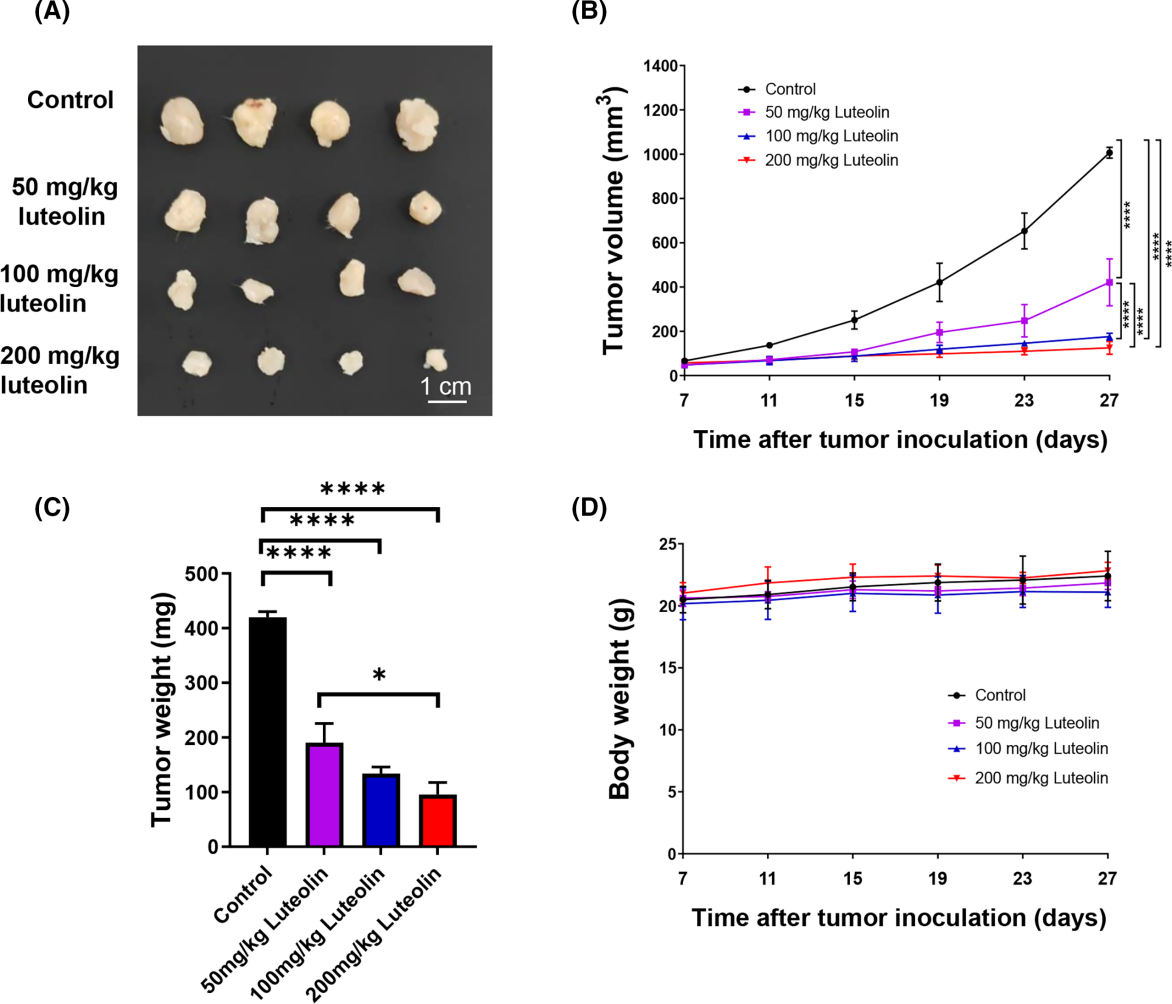
Luteolin exerts a dose‐dependent antitumor effect. Representative tumour images (A), tumour growth curves (B), tumour weight (C) and body weight (D) of H22 tumour‐bearing BALB/c mice in the control group and 50, 100 and 200 mg/kg luteolin groups. *N* = 4, data are presented as mean ± SD. One‐way ANOVA, *p < 0.01, ****p < 0.0001.

To assess the anti‐tumour effect of luteolin, we subcutaneously injected BALB/c mice with H22 cells. The tumour images (Figure 1A), tumour growth curves (Figure 1B) and tumour weight (Figure 1C) indicated that the degree of tumour malignancy considerably decreased after luteolin treatment (50, 100 and 200 mg/kg) in a dose‐dependent manner compared with that in the control group. Moreover, luteolin had no influence on body weight relative to the control (Figure 1D).

### 3.2 Multiple functions are affected by luteolin treatment

**FIGURE 2:**
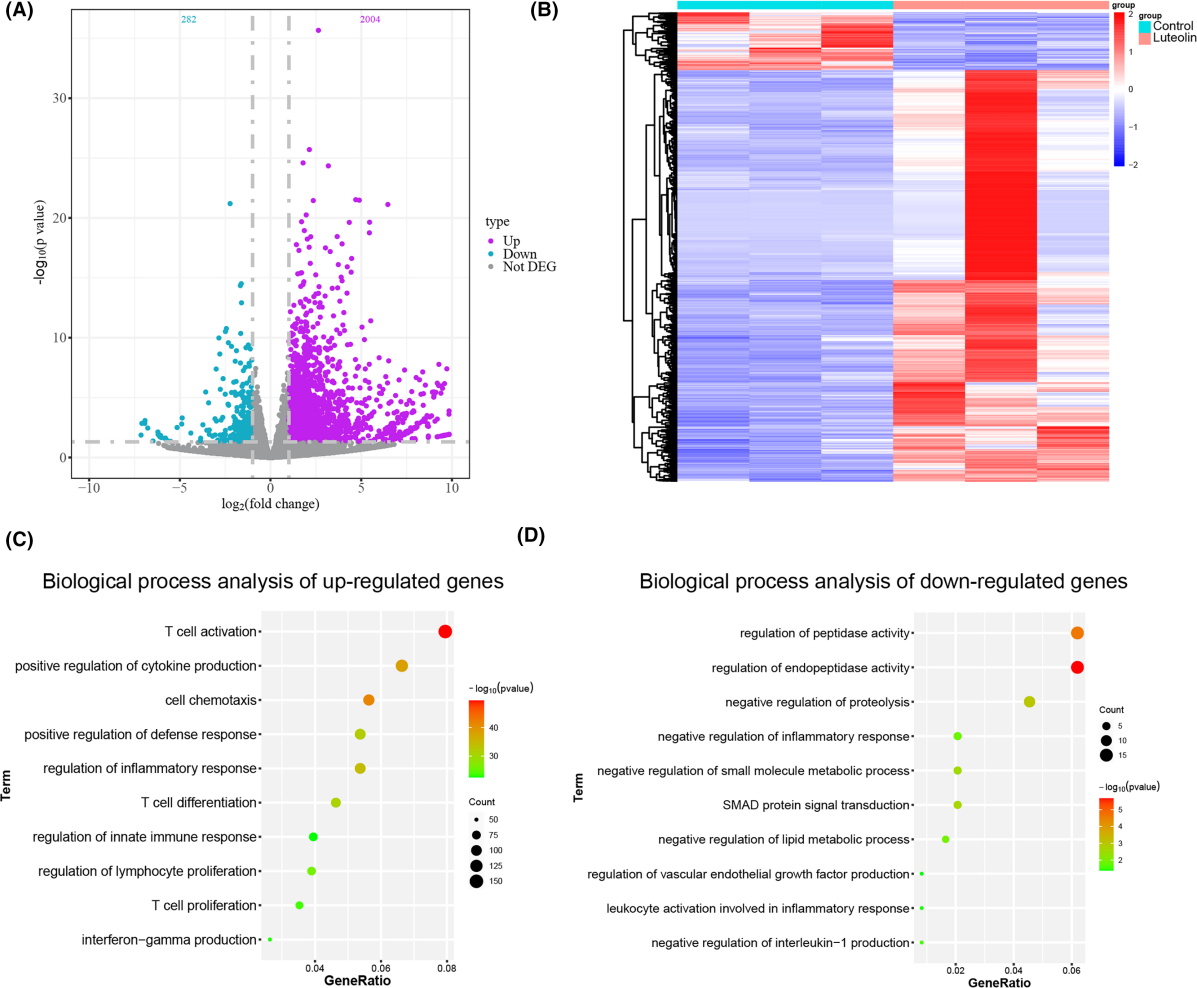
Multiple functions are affected after 200 mg/kg luteolin treatment. (A) Volcano map of the expression profile of differential genes between the control and luteolin groups. (B) Cluster analysis of the expression profile of differential genes between the control and luteolin groups. Main biological processes of up‐regulated (C) and down‐regulated genes (D).

To investigate the functions affected by luteolin treatment, we collected tumours from H22 tumour‐bearing BALB/c mice and performed high‐throughput sequencing via the NovaSeq platform (Illumina). The volcano map generated from differential genes was shown in Figure 2A. A total of 2286 differentially expressed genes, including 2004 up‐regulated genes (purple dots) and 282 down‐regulated genes (blue dots), were found. Cluster analysis distinguished the luteolin group from the control group (Figure 2B).

Gene ontology (GO) enrichment analysis revealed that the main biological processes (BPs) of the up‐regulated genes were T‐cell activation, positive regulation of cytokine production, cell chemotaxis, regulation of inflammatory response, regulation of lymphocyte proliferation and IFN‐γ production (Figure 2C). The main BPs of the down‐regulated genes were regulation of endopeptidase activity, negative regulation of inflammatory response, SMAD protein signal transduction, regulation of vascular endothelial growth factor production and negative regulation of interleukin‐1 production (Figure 2D).

We also studied mRNA related to CD8^+^ T‐cell infiltration, CD8^+^ T‐cell activation and apoptosis. After treatment with 200 mg/kg luteolin, the mRNA levels of CD8^+^ T cells infiltration (Cd3e, Cd8a, Ccl5 and Ccl21), CD8^+^ T‐cell activation (Gzmb, Ifng and Tnf) and apoptosis (Casp3, Casp8, Casp9, Bax and Bcl2) increased considerably compared with those in the control group (Figure S1A–L). These results demonstrated that luteolin can induce the inflammatory response and enhance the immune response.

### 3.3 Luteolin increases the proportion of CD8 ^+^ T lymphocytes in the spleen, peripheral blood and tumour tissues

**FIGURE 3:**
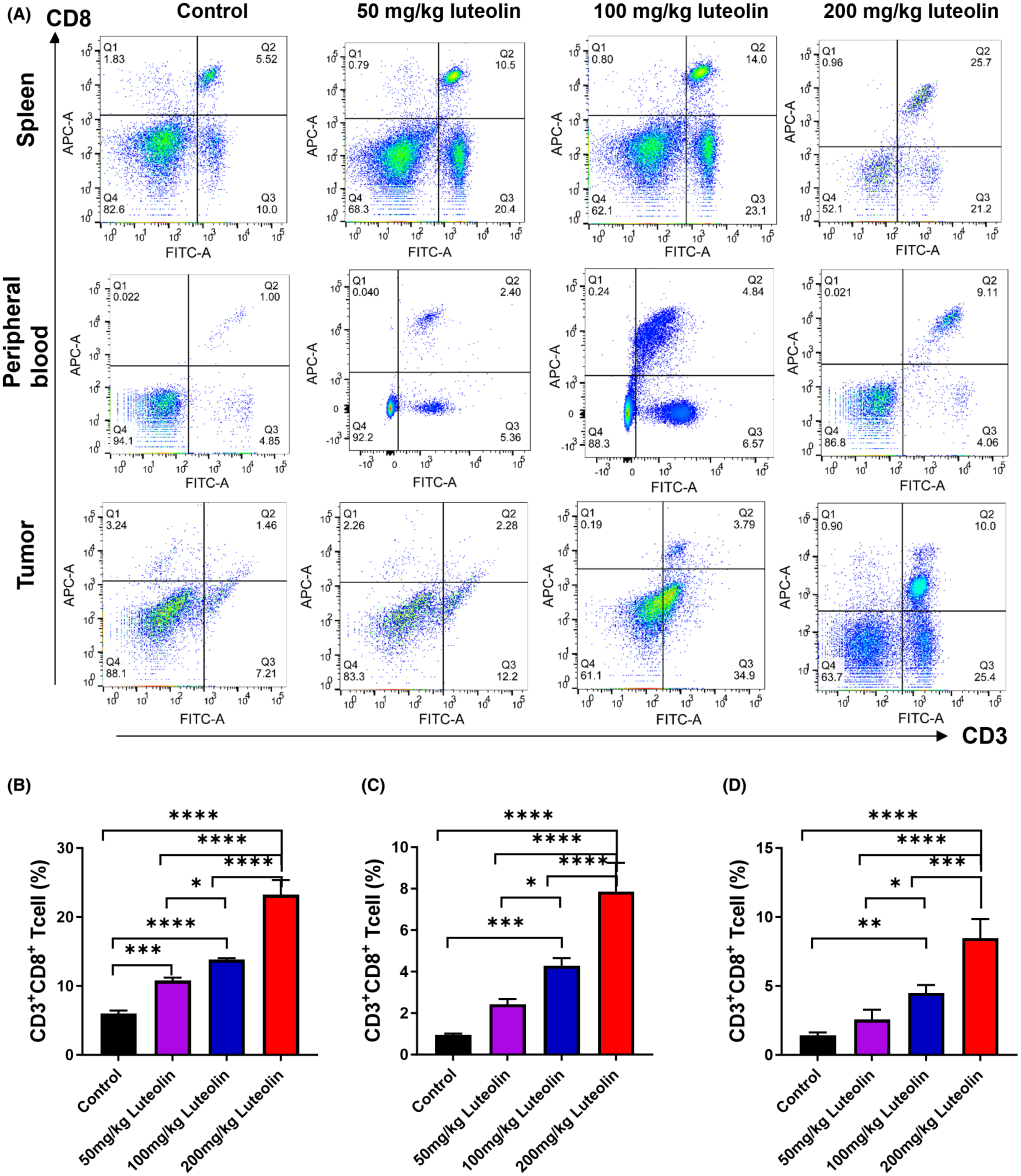
Luteolin increases the proportion of CD8^+^ T lymphocytes in the spleen, peripheral blood and tumour tissues. (A) Percentage of CD8^+^ T lymphocytes in the spleen, peripheral blood and tumour tissues of H22 tumour‐bearing mice determined by flow cytometry. Statistical analysis of the percentage of CD8^+^ T lymphocytes in the spleen (B), peripheral blood (C) and tumour tissues (D) of H22 tumour‐bearing mice. *N* = 4, data are presented as mean ± SD. One‐way ANOVA, *p < 0.05, **p < 0.01, ***p < 0.001, ****p < 0.0001.

We investigated whether luteolin can influence the number of TILs in H22 tumour‐bearing mice. We detected the percentage of CD8^+^ T lymphocytes in the spleen, peripheral blood and tumour tissues by flow cytometry (Figure 3A). With regard to the spleen (Figure 3B), peripheral blood (Figure 3C) and tumour tissues (Figure 3D), the percentage of CD8^+^ T lymphocytes was elevated in the 50, 100 and 200 mg/kg luteolin groups compared with that in the control group. These lines of evidence indicated that luteolin can remarkably increase the proportion of CD8^+^ T cells in tumour‐bearing mice and that luteolin may have the potential to boost levels of CD8^+^ T lymphocytes in vivo.

### 3.4 Luteolin promotes the infiltration and cytotoxicity of CD8 ^+^ T lymphocytes in tumour tissues

**FIGURE 4:**
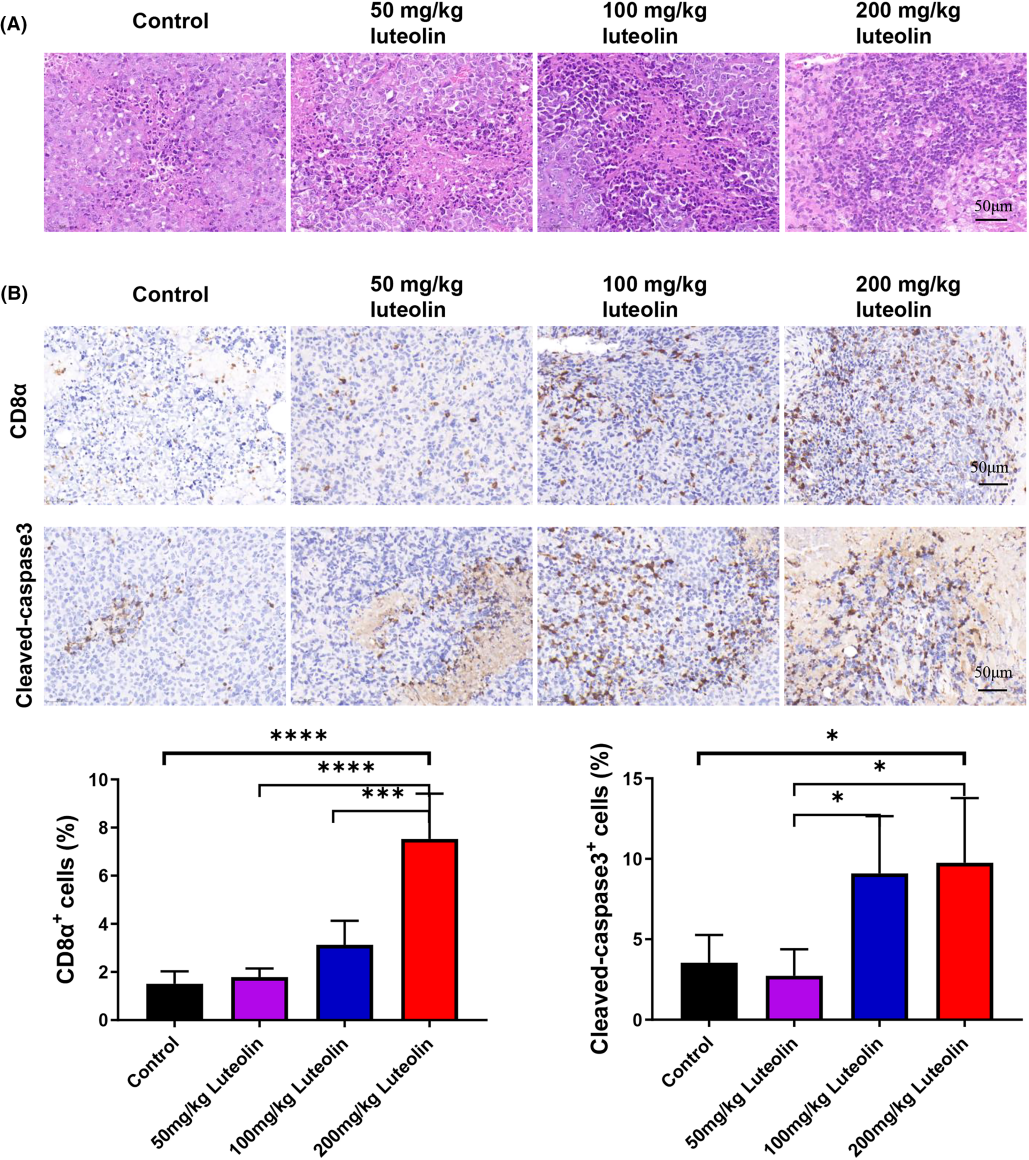
Luteolin promotes the infiltration and cytotoxicity of CD8^+^ T lymphocytes in tumour tissues. (A) Haematoxylin and eosin staining of TILs. Tumour cells are indicated by large nuclear stains and lymphocytes are represented by small nuclear stains. (B) Immunohistochemistry staining reveals the expression of CD8α and cleaved caspase‐3 in tumour tissues extracted from H22 tumour‐bearing mice. *N* = 4, data are presented as mean ± SD. One‐way ANOVA, *p < 0.05, ***p < 0.001, ****p < 0.0001. Scale bar, 50 μm.

This investigation also aimed to examine if luteolin could affect the morphology and function of TILs in H22 tumour‐bearing mice. As shown in Figure 4A, the number of TILs, particularly CD8^+^ T lymphocytes, increased in the different luteolin treatment groups compared with that in the control group. IHC staining indicated that the CD8α levels in the 50, 100 and 200 mg/kg luteolin groups were higher than that in the control group (Figure 4B). These findings suggested that luteolin treatment, particularly 100 and 200 mg/kg luteolin, can effectively induce CD8^+^ T lymphocyte infiltration into tumour tissues in H22 solid tumours. We also studied the mRNA level of chemokines related to T‐cell infiltration. After treatment with 200 mg/kg luteolin, the levels of Cd3e, Cd8a, CCL5 and CCL21, increased considerably compared with that in the control group (Figure S1A–D). The effects of luteolin on tumour apoptosis were further evaluated. IHC staining indicated higher cleaved caspase‐3 levels in the 50, 100 and 200 mg/kg luteolin groups compared with those in their control counterparts (Figure 4B). Furthermore, we analysed the correlation of the numbers of CD3^+^CD8^+^ T cells with apoptosis levels and tumour size. Spearman's correlation analysis was performed and the results revealed that the numbers of CD3^+^CD8^+^ T cells were positively correlated with apoptosis levels (*R* = 0.6711, *p* = 0.0044) and negatively correlated with tumour size (*R* = −0.9088, *p* = 0; Figure S2A,B). These findings suggested that luteolin could effectively promote the infiltration and enhance the cytotoxicity of CD8^+^ T lymphocytes within tumour tissues in H22 solid tumours.

### 3.5 Luteolin enhances the cytotoxicity of tumour‐infiltrating CD8 ^+^ T cells and affects inflammatory and effector chemokines

**FIGURE 5:**
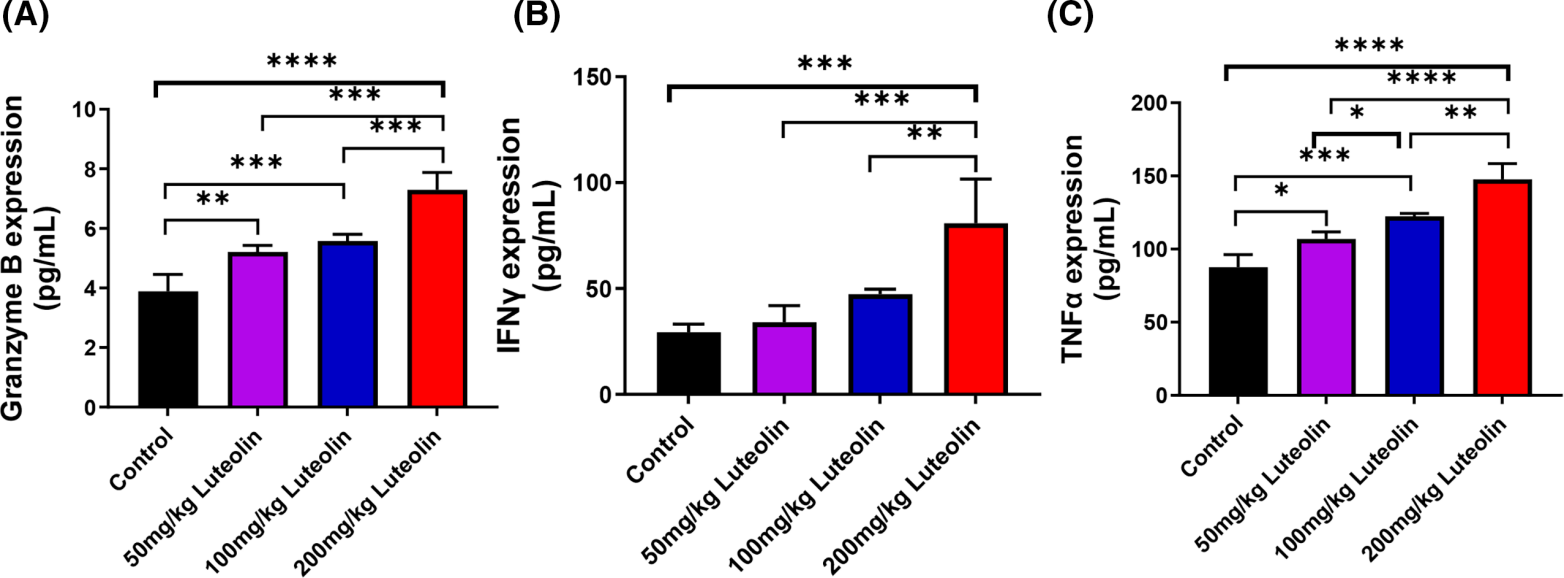
Luteolin enhances the cytotoxicity of tumour‐infiltrating CD8^+^ T lymphocytes and affects inflammatory and effector chemokines. Levels of granzyme B (A) and cytokines, such as IFN‐γ (B) and TNF‐α (C), in peripheral blood are measured using the mouse ELISA kit (*N* = 4, data are presented as mean ± SD). One‐way ANOVA, *p < 0.05, **p < 0.01, ***p < 0.001, ****p < 0.0001.

GO analysis revealed that luteolin could promote T‐cell activation and positively regulate cytokine production. CD8^+^ T lymphocytes could directly destroy cancer cells by releasing cytotoxic granules, such as granzyme B and cytokines, such as IFN‐γ and TNF‐α. We studied the protein level of granzyme B, IFN‐γ and TNF‐α via ELISA. The immune response was enhanced, as indicated by the increased secretion of granzyme B (Figure 5A), IFN‐γ (Figure 5B) and TNF‐α (Figure 5C) after luteolin treatment relative to that in the control. These data suggested that luteolin might activate the function of CD8^+^ T lymphocytes.

### 3.6 Luteolin enhances the anti‐tumour effect of the PD‐1 inhibitor on H22 tumour‐bearing BALB/c mice

**FIGURE 6:**
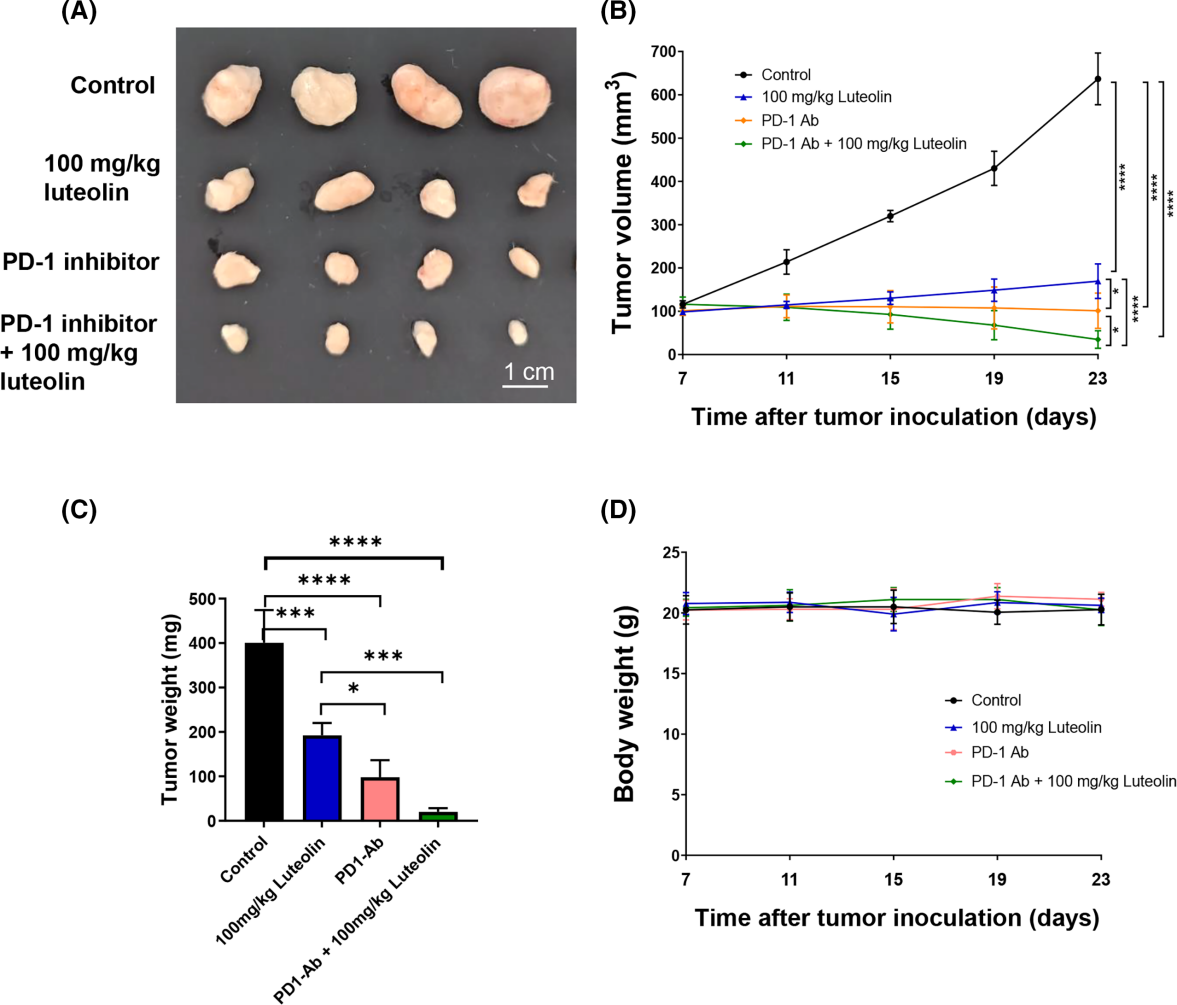
Luteolin enhances the antitumor effect of the PD‐1 inhibitor on H22 tumour‐bearing BALB/c mice. Representative tumour images (A), tumour growth curves (B), tumour weight (C) and body weight (D) of H22 tumour‐bearing mice after luteolin and PD‐1 inhibitor (alone or in combination) treatment (*N* = 4, data are represented as mean ± SD). One‐way ANOVA, *p < 0.05, ***p < 0.001, ****p < 0.0001.

Given that luteolin increased the number of CD8^+^ T lymphocytes in the spleen, peripheral blood and tumour tissues of H22 tumour‐bearing mice, we hypothesize that luteolin can enhance the anti‐tumour effect of the PD‐1 inhibitor. Therefore, the anti‐tumour effects of luteolin and the PD‐1 inhibitor were evaluated using BALB/c mice engrafted with H22 cells via subcutaneous injection. The tumour images (Figure 6A), tumour growth curves (Figure 6B) and tumour weight (Figure 6C) collectively substantiated the inhibitory effect of luteolin and the PD‐1 inhibitor on tumour growth. Moreover, the combination of luteolin and the PD‐1 inhibitor exhibited superior anti‐tumour efficacy compared with luteolin or the PD‐1 inhibitor alone. No influence on body weight was observed after treatment with luteolin or the PD‐1 inhibitor (Figure 6D). These results indicated that luteolin could enhance the anti‐tumour effects of the PD‐1 inhibitor.

## 4 DISCUSSION

The efficacy of traditional Western medicine remains unsatisfactory due to the complexity of the carcinogenesis and development of HCC. Increasing interest has been directed toward exploring the multitarget treatment characteristics of traditional Chinese medicine (TCM). Although TME and its interaction with TCM have gained attention, studies that focused on the effect of TCM on CD8^+^ T lymphocyte infiltration are relatively limited. The current research addresses this gap by comprehensively investigating the infiltration, phenotype, killing capacity, chemokines and cytokine secretion related to CD8^+^ T lymphocytes.

Luteolin has potent anti‐tumour properties against various human malignancies; examples of these properties include the inhibition of tumour cell proliferation, protection against carcinogenic stimuli, induction of cell cycle arrest and induction of apoptosis through distinct signalling pathways.^10^ Our previous study demonstrated that luteolin has direct inhibitory properties against HCC growth.^25^ Jiang et al.^24^ revealed that luteolin suppresses inducible PD‐L1 expression to improve anti‐tumour immunity in KRAS‐mutant lung cancer. The current work found that luteolin has the potential to stimulate an immune response against tumours by promoting the infiltration of CD8^+^ T lymphocytes.

Immune infiltrates within tumours, often referred to as TILs, have been increasingly recognized as a critical factor in the search for optimal biomarkers.^26^ TILs are a type of mononuclear immune cells that penetrate tumour tissues. The presence of TILs provides insight into an individual's immunological response status and holds promise for clinical applications.^27^ Recent studies highlighted the critical roles of TILs in TME and their effect on tumour progression and prognosis.^28^, ^29^, ^30^, ^31^, ^32^ Wang et al.^33^ revealed that CTLA‐4 blockade triggers tumour cell pyroptosis via the release of IFN‐γ and TNF‐α from activated CD8^+^ T lymphocytes. The secretion of these cytokines mediates cytotoxicity and enhances the overall anti‐tumour immune response. In the current study, we found that these cytokines were elevated at the gene and protein levels in the luteolin group.

An increasing number of preclinical and clinical trials have revealed that treatments that combine immune inhibitors and other therapies can yield superior anti‐tumour efficacy relative to monotherapy.^34^, ^35^ This result is consistent with our finding that the combination of luteolin and the PD‐1 inhibitor enhanced the anti‐tumour effect on the HCC model.

## 5 CONCLUSION

**FIGURE 7:**
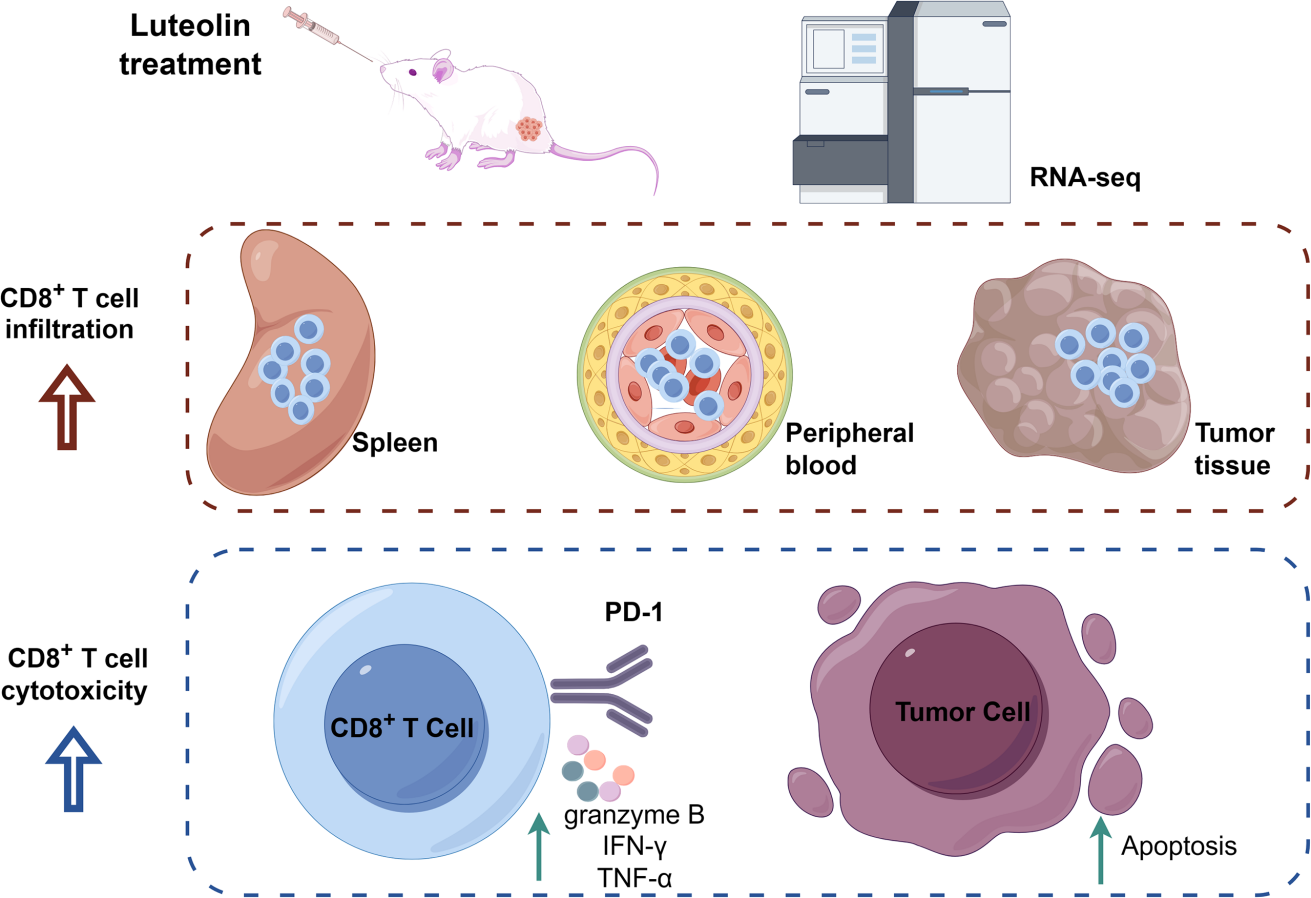
Graphical abstract of this study.

Our study demonstrated that luteolin effectively inhibited tumour growth in H22 tumour‐bearing mice; sustained high levels of CD8^+^ T lymphocytes in the spleen, peripheral blood and tumour; and boosted their cytotoxicity in tumour tissues. These lymphocytes also showed increased activation and cytokine production. The combined administration of luteolin and the PD‐1 inhibitor enhanced the anti‐tumour effects on H22 tumour‐bearing mice (Figure 7).

## AUTHOR CONTRIBUTIONS

**Shijiao Cai:** Data curation (equal); writing – original draft (equal). **Yidan Gou:** Data curation (equal); writing – original draft (equal). **Yanyan Chen:** Methodology (supporting). **Xiaoran Hou:** Methodology (supporting). **Jing Zhang:** Methodology (supporting). **Chongwen Bi:** Data curation (supporting). **Peng Gu:** Data curation (supporting). **Miao Yang:** Data curation (supporting). **Hanxu Zhang:** Data curation (supporting). **Weilong Zhong:** Project administration (equal). **Hengjie Yuan:** Project administration (equal).

## CONFLICT OF INTEREST STATEMENT

The authors declare no conflicts of interest.

## Supporting information


Figure S1.

Figure S2.

Table S1.

## Data Availability

All datasets used and/or analysed during the current study are available from the corresponding author on reasonable request.
