# Supplementary material for: Luteolin exerts anti‐tumour immunity in hepatocellular carcinoma by accelerating CD8 + T lymphocyte infiltration
Source: J Cell Mol Med. 2024 Sep 12;28(17):e18535. doi: 10.1111/jcmm.18535 (PMC11392827; doi:10.1111/jcmm.18535)
Supplement: Supplementary file 1 — Figure S1. [file JCMM-28-e18535-s002.docx]

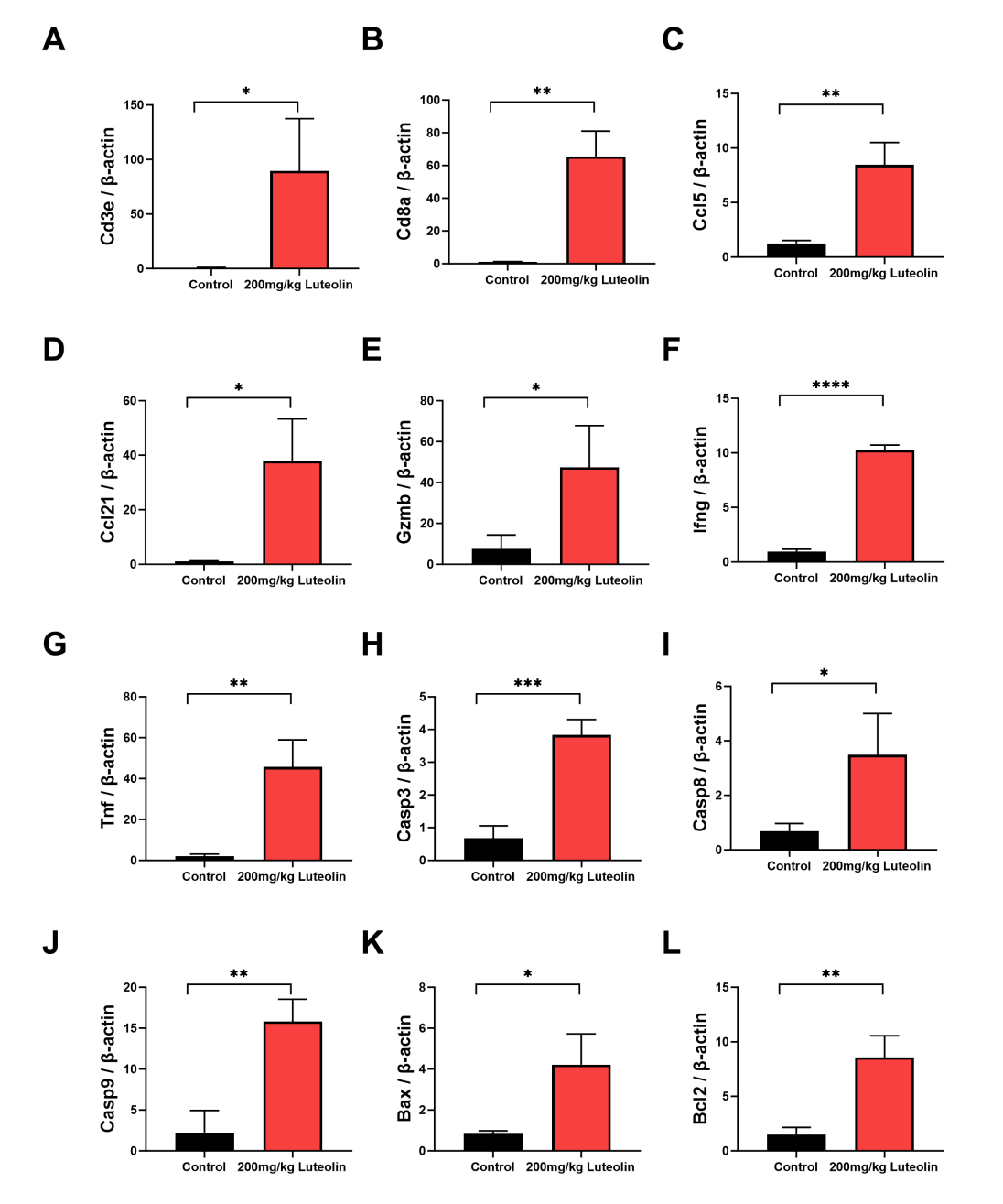


Supplemental figure 1. CD8^+^ T cell infiltration, CD8^+^ T cell activation, and apoptosis related-mRNA in tumors from control and 200 mg/kg luteolin groups were detected by qRT-PCR. (A) CD3e, (B) CD8a, (C) Ccl5, (D) Ccl21, (E) Gzmb, (F) Ifng, (G) Tnf, (H) Casp3, (I) Casp8, (J) Casp9, (K) Bax, and (L) Bcl2 were normalized to β-actin. Data are means ± SD (n = 3).
