# Supplementary material for: Luteolin exerts anti‐tumour immunity in hepatocellular carcinoma by accelerating CD8 + T lymphocyte infiltration
Source: J Cell Mol Med. 2024 Sep 12;28(17):e18535. doi: 10.1111/jcmm.18535 (PMC11392827; doi:10.1111/jcmm.18535)
Supplement: Supplementary file 2 — Figure S2. [file JCMM-28-e18535-s001.docx]

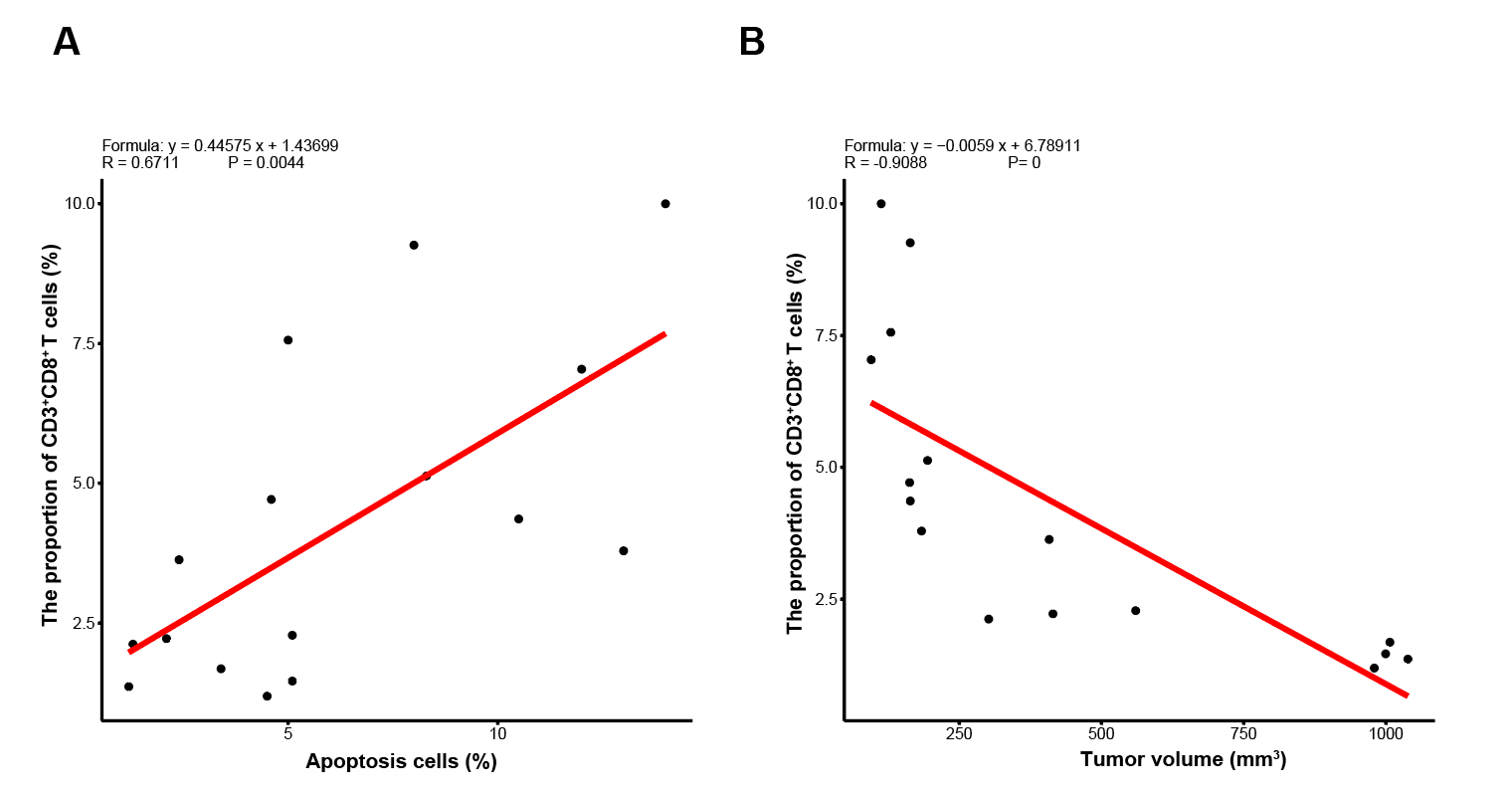


Supplemental figure 2. Correlation between the numbers of CD3^+^CD8^+^ T cells and tumor conditions, such as apoptosis level and tumor size, obtained by Spearman’s analysis of the control, 50 mg/kg luteolin, 100 mg/kg luteolin, and 200 mg/kg luteolin groups.
