# Supplementary material for: Luteolin exerts anti‐tumour immunity in hepatocellular carcinoma by accelerating CD8 + T lymphocyte infiltration
Source: J Cell Mol Med. 2024 Sep 12;28(17):e18535. doi: 10.1111/jcmm.18535 (PMC11392827; doi:10.1111/jcmm.18535)
Supplement: Supplementary file 3 — Table S1. [file JCMM-28-e18535-s003.docx]

**Supplemental Table 1**

Primers used for real-time quantitative reverse transcription PCR

| Gene | Sequence |
| --- | --- |
| Casp3  Casp8  Casp9  Bax  Bcl2  Cd3e  Cd8a  Ccl5  Ccl21  Gzmb  Ifng  Tnf  β-Actin | F: 5′-GAGCTGGACTGTGGCATTGA-3′  R: 5′-GAACCACGACCCGTCCTTT-3′  F: 5′-AGACTGCAACCGAGAGGAGA-3′  R: 5′-CGCTCACTTCTTCTGAGAGC-3′  F: 5′-TGCCCTTGCCTCTGAGTAGT-3′  R: 5′-AACAAAGAAACGCCCACAAC-3′  F: 5′-CTCAAGGCCCTGTGCACTAA-3′  R: 5′-GGAGAGGAGGCCTTCCCAG-3′  F: 5′-CCCTGTGGATGACTGAGTACC-3′  R: 5′-CTTCAGAGACAGCCAGGAGAAA-3′  F: 5′-GTGTAGAGTTGACGTGCCCT-3′  R: 5′-AGTAGCCACTGTCCTCGACT-3′  F: 5′-CGGATTGGACTTCGCCTGT-3′  R: 5′-CTAGCGGCCTGGGACATTTG-3′  F: 5′-GTGCCCACGTCAAGGAGTAT-3′  R: 5′-CTCTGGGTTGGCACACACTT-3′ |
|  | F: 5′-CCCTACAGTATTGTCCGAGGC-3′ |
|  | R: 5′-GTGAGAACAGGATTGCCGGG-3′ |
|  | F: 5′-GAAGCCAGGAGATGTGTGCT-3′ |
|  | R: 5′-GCACGTTTGGTCTTTGGGTC-3′ |
|  | F: 5′-AAGACAATCAGGCCATCAGC-3′ |
|  | R: 5′-CTGGACCTGTGGGTTGTTGA-3′ |
|  | F: 5′-CCTCACACTCACAAACCACCA-3′ |
|  | R: 5′-ACAAGGTACAACCCATCGGC-3′ |
|  | F: 5′-CCTCACTGTCCACCTTCC-3′ |
|  | R: 5′-GGGTGTAAAACGCAGCTC-3′ |
